# Maternal milk in the NICU: An everyday intervention to improve brain development

**DOI:** 10.1038/s41390-024-03125-3

**Published:** 2024-03-04

**Authors:** Stephanie L. Merhar, Steven P. Miller

**Affiliations:** 1 Perinatal Institute, Division of Neonatology, Cincinnati Children’s Hospital Medical Center, Cincinnati, Ohio and Department of Pediatrics, University of Cincinnati, Cincinnati, OH USA; 2 Department of Pediatrics, BC Children’s Hospital Research Institute and the University of British Columbia, Vancouver, BC Canada

Despite improvements in mortality after preterm birth due to advancements in neonatal intensive care, very preterm infants (born <32 weeks gestation) still experience significant morbidity, including neurodevelopmental and behavioral problems.^1^ It has long been known that poor growth is a risk factor for worse neurodevelopmental outcomes in preterm infants.^2,3^ The ideal nutrition source for preterm infants is maternal breast milk fortified with additional macronutrients.^4^ There is evidence that maternal milk improves many preterm morbidities and is associated with improved brain development as seen on MRI and higher scores on neurocognitive tests at preschool and at school age.^5^ However, it is unknown which particular factors in human milk lead to the components of brain health that underlie these better neurodevelopmental outcomes.

Lactoferrin is the main protein present in human milk.^6^ It has antimicrobial and anti-inflammatory properties and has been shown to reduce the incidence of common morbidities of prematurity such as sepsis.^7^ Lactoferrin can improve cognition and memory in animal models.^8^ These improvements may be due to protection from neuronal injury, enhanced neurotrophin production, and decreased inflammation. Studies on the effects of lactoferrin specifically on preterm human brain development have thus far been lacking.

To address this knowledge gap, Atayde et al studied 36 infants born at <32 weeks gestation who were fed human milk and had brain MRI at term equivalent age.^9^ In this observational cohort, they measured lactoferrin concentrations in milk at 14 and 28 days of age and compared outcomes in the infants exposed to low lactoferrin versus high lactoferrin. After adjustment for sex, gestational age at birth, birth weight z-score, and postmenstrual age at MRI, they found that infants in the top tertile of lactoferrin exposure had larger brain volumes, cortical gray matter, and deep gray matter at term equivalent age. As lactoferrin is highly correlated with protein, they did an additional analysis adjusting for protein intake and found that the increase in deep gray matter volume remained statistically significant. In this adjusted analysis, infants exposed to higher lactoferrin also had larger cerebellar volumes.

There is a pressing need to understand how nutrition and nutritional interventions affect the preterm brain, and work such as this is an important step. When considering brain health in the preterm newborn, attention is shifting away from brain injury as a static single “incident” with fixed consequence. Even white matter injury, the prevalent pattern of brain injury in the preterm neonate, is characterized by white matter dysmaturation – impaired maturation of the oligodendroglia lineage.^10^ Numerous studies point to the importance of the trajectory of brain maturation that evolves over time as the critical determinant of neurodevelopmental outcome.^11,12^ Importantly, brain maturation evolves through the period of neonatal intensive care and beyond. This reveals a window of opportunity for beneficial interventions, including nutrition.^13^

Numerous lines of research evidence now point to the *importance of everyday* practices for long-term brain maturation in preterm neonates. These relationships are increasingly recognized to have relative structural specificity in the brain. For example, during weeks to months in the neonatal intensive care unit (NICU), preterm neonates undergo many painful procedures as part of life-saving care. Pain is a key predictor of slower neonatal thalamic development which is in turn associated with neurodevelopmental outcome.^14^ The medications used to provide analgesia or sedation, including benzodiazepines such as midazolam, are also linked to specific alterations in brain maturation. For example, impaired *neonatal* hippocampal growth is related to NICU midazolam exposure with *persisting* hippocampal abnormalities to 8 years of age; these differences in hippocampal development are associated with specific aspects of cognition.^15,16^ These regionally specific associations add another dimension to multivariable modelling when trying to disentangle complex NICU exposures in observational studies.

Nutrition is another critical everyday exposure that is related to specific aspects of brain maturation. For example, nutritional intake in the first 2 weeks of life predicts brain maturation to term equivalent age.^13^ Other studies have focused on more specific aspects of early nutrition which may enhance brain development, such as long chain fatty acids^17,18^ and even more specifically docosahexaenoic acid.^19^ Importantly, better nutrition modifies the association between other aspects of neonatal adversity (e.g. mechanical ventilation) on brain growth.^13^ The findings of Atayde et al focus on a specific aspect of breast milk that requires attention.

In this article, lactoferrin was associated with better overall brain growth. Infants with higher lactoferrin exposure had larger overall brain volumes as measured by MRI at term equivalent age. As lactoferrin is the major protein in human milk, it may be the increased protein levels themselves that are helpful for brain growth. There is a large body of literature suggesting that protein is key for early growth and mitigation of brain injury.^20,21^ Enteral protein intake in particular (versus parenteral protein intake from TPN) appears to be highly linked to brain growth.^22^ The mechanisms relating lactoferrin exposure to improved brain growth are not well understood, but there are several plausible mechanisms. Decreased infection and inflammation due to the antimicrobial and anti-inflammatory effects of lactoferrin could have positive effects on brain development. Lactoferrin is an iron-binding agent and can exist in the apo (iron-free) or holo (iron-saturated) state, both of which are observed in human milk.^6^ The apo state can chelate iron and act as an antibacterial agent while the holo state can correct iron deficiency; decreased infections and increased iron stores are both positively associated with brain development. Studies with a larger sample size and the ability to include more variables in the statistical model might allow for further elucidation of the possible neuroprotective mechanisms of lactoferrin (e.g. overall protein, infection/inflammation, iron status).

Lactoferrin was also associated with larger volumes of cortical gray matter and deep gray matter. Again, overall protein intake in the first 28 days of life has been associated with increased deep gray matter volumes at term equivalent age,^23^ so it may be overall protein and not specifically lactoferrin that is important. However, when adjusting for overall protein intake, lactoferrin was still associated with larger deep gray matter volumes at term equivalent. Substantial gray matter volume growth occurs during the third trimester, so it may be that the brain regions with the highest growth rate in the third trimester are the ones most affected by nutritional interventions during this period. This is especially relevant clinically given the important role of the basal ganglia in the cognitive outcomes of preterm children.^24^ When adjusting for overall protein, cerebellar volumes were also higher in the high lactoferrin group. The cerebellum enlarges fivefold between 24 weeks of gestation and term, and during this time also forms connections to the cortex and subcortical structures. In one study including 67 preterm infants, cumulative lipid and energy intake in the first 4 weeks of life were associated with significantly greater cerebellar volume at term equivalent age.^25^ In another of 60 preterm neonates, higher near-birth docosahexaenoic acid levels were associated with larger cerebellar volumes near term.^18^ Together, these studies highlight the importance of nutrition on cerebellar development.

Nutrition is a complex intervention and brain development is a complex process. We should not assume that nutritional interventions, even if provided at similar brain developmental stages, will affect the whole brain equally. Studies such as this, pairing sensitive analysis of specific nutritional substances with advanced quantitative neuroimaging techniques, allow for a better understanding of the complex interplay between nutrients and specific areas of brain development. This study adds to the body of literature on the potential mechanisms of neuroprotection of human milk in preterm infants and the importance of routine everyday interventions such as nutrition to the trajectory of brain health in children born preterm.
